# Liebreich on Ozone

**Published:** 1881-06

**Authors:** 


LIEBREICH ON OZONE.

We know that ozone had originally a very hard time as a chemical substance; chemists first of all denied its existance, and later on, undervalued it. It first obtained its due place among chemical snbstances by the experimental researches of Schönbein and his followers. The fate of ozone in chemistry will probably not be repeated in therapeutics, as we have here to deal with a substance which is already well known beforehand, and we know the action which ozone can exert on the organism. We possess a substance which is extraordinarily like ozone in the peroxide of hydrogen, which formerly belonged to those substances that, although described and brought forward in books and learned societies, were rarely seen and then only by a few learned persons. We have not to thank any scientific efforts that it now has become a matter of general knowledge, but only researches for cosmetic purposes.

Peroxide of hydrogen was first manufactured on a large scale for the purpose of dyeing dark hair yellow, and through this technical application it has become possible to experiment with this substance in large quantities. We know that it possesses quite opposite and peculiar properties, both of conservation and destruction. Liebreich has on another occasion stated that milk can, for example, be preserved for a long time with peroxide of hydrogen without undergoing putrefaction, and that, on the other hand, blood immediately decomposes peroxide of hydrogen. When peroxide of hydrogen is put into a wine glass, and a teaspoonful of blood is added, it foams like champagne, not with carbonic acid gas, but merely with oxygen. It was naturally an easy step to repeat this result on the animal organism. Liebreich accordingly entrusted Dr. Schwerin with this research; and Dr. Guttmann at the same time undertook independently a similar investigation, when the remarkable interesting result was attained, that the decomposition is effected in the same manner in the animal organism as outside the body.

This is one of the most surprising pharmacological experiments that can be made. When peroxide of hydrogen is injected into a dog, the decomposition actually takes place in the subcutaneous connective tissue, and the animal is correspondingly distended at these places; a local emphysema is excited by the giving off of the combined oxygen in small quantities; this result only is attained, and no general effects are produced. When, however, considerable quantities are continuously injected into the animal, certain quantities of the peroxide of hydrogen are absorbed. It might be anticipated that, when a substance is injected which supplies oxygen to the blood, the animal would find itself in the very excited condition which is observed when an animal is introduced into an atmosphere of oxygen. It is, however, characteristic in all the processes of nutrition that substances are not in themselves advantageous to the organism, except in their right places; and thus we see this vital gas, when introduced by a substance which is from the tissues of the body, become a poison to the organism, the animal falls dead, and when a post-mortem examination is made, it is found on dissection that the veins are bright red, and, above all, in the blood are distributed bubbles of oxygen. The blood does not take up the oxygen; it has indeed been absorbed there and then on the spot, but a coagulation of blood is produced by the breaking up of the peroxide of hydrogen, which supplies more oxygen than it needed; the blood vessels are obstructed, and the animal dies with coagulation of the blood, suffocated, while possessing an excess of the oxygen, which, if taken in by the lungs, would have been effective in the opposite direction. Thus, then, it is seen that peroxide of hydrogen and the oxygen, produced by it internally, are of no advantage to the animal. By these researches, the hopes which have been entertained of the therapeutic value of peroxide of hydrogen are annihilated. It is not possible to introduce into the organism any useful supply of oxygen by the internal administration of this substance, peroxide of hydrogen, so rich in that life-giving gas. The same holds good with ozone, with the difference, however, that the latter, when it has been employed, up to the present time has not been experimentally tested, and that therapeutic records supply statements which are directly opposed to the scientifically investigated phenomena of ozone.

The first error began with the fact that people are in the habit of speaking in general terms of the contents of ozone, and that various balneologists speak of an atmosphere rich in ozone. We do not know what is meant by rich in ozone or poor in ozone, since we cannot measure it; and in such a phraseology, desires appear to be confounded with powers. We know that ozone behaves itself just like other oxidizing substances; iodide of potassium, as is generally known, can be mixed with starch, and in this way white iodized starch paste be produced; when blotting paper is impregnated with this, we obtain a white reäctive paper, and when this is moistened with an oxidizing solution, whether it be of chlorine or of nitrous acid, or any other oxidizing solution, iodine is set free, and it is known that the starch is colored blue by the combination of the particles of iodine and starch; all oxidizing substances produce these appearances. This iodized starch paper, when hung out in a still atmosphere, may be subjected to an atmosphere very rich in ozone, and yet the paper be but very slightly colored blue; when, on the other hand there is a rapid movement of the air sweeping over a paper, although the air may have a much smaller amount of ozone, the paper will nevertheless become much more intensely blue. From the blue color obtained under such circumstances, no practical result can be deduced. No value can be attached to any observations, unless it is known how much air in a given time has passed over the paper.

Another question is this: Can ozone exercise any therapeutic effect? This question, in the sense in which it is now put forward, is one which must be answered in the negative. Ozone occupies the same space, according to volume, as oxyen gas. Thus, when a physician says to himself, 'I have a given quantity of air which contains 32 parts in weight of oxygen, the same contains in equal volume 48 parts of ozone,' any one who relies on this simple fact, and does not examine the physiology of the case, may say to a physician, 'This must evidently be a very valuable means of introducing oxygen into the circulation, since when an individual breathes, he has, in a given quantity of atmosphere, breathed one-third more oxygen that in a normal atmosphere.' This would be so far correct, if ozone resembled entirely oxygen in its external properties. Ozone is, however, an extremely labile body, which, in contact with the fluid tissues, immediately decomposes and gives off oxygen. This free oxygen is in a nascent state, which oxidizes very rapidly, and it acts when it is given off externally on the tissues, just like diluted chlorine or any other oxidizing agent. When we, therefore, inhale ozone, it becomes completely decomposed in the mucous membrane of the mouth and in the trachea; it acts as an irritant, and it is observable that concentrated quantities of ozone give rise to the same phenomena of irritation. How can we, when we know certainly of such a substance, that it is, at the outset destroyed, assume that it has a therapeutic action? We have no means at present by which we can introduce ozone into the circulation, and, if we were to do so, the same phenomena would result as when we introduce peroxide of hydrogen; oxygen would be given off, bladders full of oxygen would be formed which would produce suffocation. Blood, it may be said, ozonizes and the blood contains ozone. Although, however, ozone may be developed from a substance, it is not correct to draw the conclusion that it is necessary for the existence of this subject. Whenever oxygen is required, we see a power of ozonizing. We find, therefore, that ozone is a product of decomposition; we find (and this is a known fact) that where decomposition occurs, ozone is present. In these few words, Liebreich aims at putting forward especially the fact that we do not posses any knowledge as to the measurements of the proportions of ozone, and that therefore no therapeutic action can be assigned to it. He does not, therefore, further enter upon alleged results attained by the use of ozone.—*London Med. Record.*

